# Homotransfer FRET Reporters for Live Cell Imaging

**DOI:** 10.3390/bios8040089

**Published:** 2018-10-11

**Authors:** Nicole E. Snell, Vishnu P. Rao, Kendra M. Seckinger, Junyi Liang, Jenna Leser, Allison E. Mancini, M. A. Rizzo

**Affiliations:** Department of Physiology, University of Maryland School of Medicine, 660 W Redwood St/HH525B, Baltimore, MD 21201, USA; nicole.snell@umaryland.edu (N.E.S.); vishnu-prak.rao@som.umaryland.edu (V.P.R.); kendra.seckinger@gmail.com (K.M.S.); jliang@som.umaryland.edu (J.L.); jleser@umaryland.edu (J.L.); a.mancini@umaryland.edu (A.E.M.)

**Keywords:** FRET, anisotropy, GFP, fluorescent protein, biosensor, homotransfer

## Abstract

Förster resonance energy transfer (FRET) between fluorophores of the same species was recognized in the early to mid-1900s, well before modern heterotransfer applications. Recently, homotransfer FRET principles have re-emerged in biosensors that incorporate genetically encoded fluorescent proteins. Homotransfer offers distinct advantages over the standard heterotransfer FRET method, some of which are related to the use of fluorescence polarization microscopy to quantify FRET between two fluorophores of identical color. These include enhanced signal-to-noise, greater compatibility with other optical sensors and modulators, and new design strategies based upon the clustering or dimerization of singly-labeled sensors. Here, we discuss the theoretical basis for measuring homotransfer using polarization microscopy, procedures for data collection and processing, and we review the existing genetically-encoded homotransfer biosensors.

## 1. Introduction

Fluorescent proteins (FPs) form the backbone of modern intracellular biosensors. Genetic encoding permits utilization of the natural protein expression machinery to allow cell-type specific labeling, even in whole organisms. Fusion to biosensing protein domains also enables the reporting of dynamic subcellular events, such as changes in second messenger concentrations or post-translational modifications. Such biosensors are designed to modulate either the intensity of a single fluorescent protein or energy transfer between two different fluorophores. Each of these design strategies has different strengths. Intensity-modulated sensors are optimal for reporting the patterns of rapidly changing phenomena, such as action potential-driven calcium changes in neurons [1]. Ratiometric sensors are well suited for quantification, and they have been used to measure the calcium concentration in vascular smooth muscle [2]. The strengths and weaknesses of these two design strategies have been reviewed extensively elsewhere [3,4,5,6,7]. We will highlight recent advances for a subtype of ratiometric biosensors that incorporate homotransfer and are advantageous for live cell experimentation.

The two fluorescent protein design-strategy relies on transferring energy from the excited state of the donor fluorescent protein to the unexcited acceptor fluorescent protein, which is then free to emit a photon. Energy migration proceeds through the Förster resonance energy transfer (FRET) mechanism. Importantly, FRET efficiency is highly sensitive to both the separation distance and the relative orientation of the FRET pair [8,9,10]. Distance changes as small as one nm can substantially affect the observed amount of energy transfer [11,12]. Thus, FRET is especially well-suited for reporting the protein conformation changes that occur during biosensor activation.

Differently colored donor and acceptor fluorophores are typically incorporated into FRET-based biosensors [13,14]. Such heterotransfer FRET designs use a blue-shifted donor, such as a cyan fluorescent protein (CFP), paired with a red-shifted acceptor like a yellow fluorescent protein (YFP). FRET can then be quantified by exciting the donor and measuring the ratio of donor and acceptor fluorescence. While this strategy has proven robust, the broad spectra of fluorescent proteins can limit some applications. First, it is difficult to pair two-color sensors with secondary sensors or optical tools such as channelrhodopsins (ChR). Second, both the donor and acceptor emission spectra of the FRET pair overlap, thereby hindering quantification and requiring the use of special illumination conditions to avoid crosstalk [15], or corrective algorithms to account for bleed-through of the donor fluorescence into the acceptor channel [16,17,18].

Both of these issues can be resolved by re-engineering two-color sensors to incorporate fluorescent proteins of the same color. FRET between two identical fluorescent species, known as homotransfer, was first observed in the early 20th century [19,20], well before the application of two-color FRET to experimental biology in the late 1960s [11]. The principles of FRET theory, including the dependence of energy transfer efficiency on the separation distance and the relative orientation of the FRET pairs, were first derived from homotransfer measurements between fluorescein molecules in a concentrated glycerol solution [21,22].

Current methods of homotransfer FRET measurement use polarized light to separate photons from the donor and acceptor fluorophores [23,24]. Polarization microscopy is particularly beneficial for quantification because it can be measured much more precisely than fluorescence intensity [23,25,26,27,28]. Here, we will discuss the use of polarized light to perform homotransfer FRET measurements and recent developments in homotransfer biosensors for biological experimentation in living cells.

## 2. Fluorescence Polarization and FRET

### 2.1. Photoselection during Fluorescence Illumination

**Figure 1:**
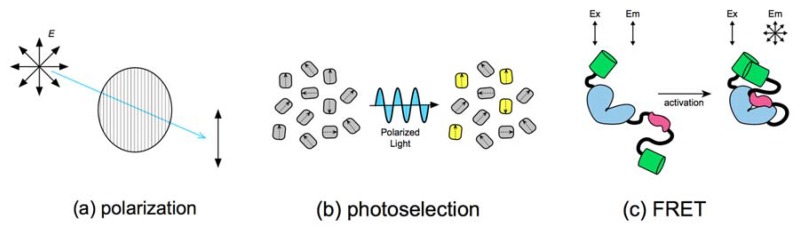
(**a**) Plate polarizers are used to constrain the illumination light to a single orientation; (**b**) polarized light can only stimulate absorption in fluorophores with compatible molecular geometries; (**c**) fluorescent proteins illuminated with polarized light will emit light in the same polarization plane. Photons emitted from Förster resonance energy transfer (FRET) excited fluorophores come from alternate orientations, depolarizing fluorescence as a function of FRET efficiency.

The fluorescence illumination must first be linearly polarized to measure homotransfer FRET using fluorescence polarization microscopy. Light waves have oscillating electric and magnetic fields that arise perpendicularly to each other. The extent that an electric field oscillates with spatial uniformity is its polarization. The modern convention is to describe planar polarization relative to the orientation of the light’s electric field. Linear polarizers that restrict the electric fields to a single direction (Figure 1a) are the primary type used for polarization microscopy.

The absorption of light by organic fluorophores is selective for specific light polarizations [29]. Since light absorption involves the physical displacement of an electron from one orbital to another, it is highly dependent on the geometry of the photon’s electric field, in addition to its energy. Thus, the illumination of a solution of randomly oriented fluorophores with polarized light will only excite a small subset of molecules that can accommodate the orientation of the light wave. This phenomenon is known as photoselection (Figure 1b) [30].

The extent that emission retains its polarization during photoselection is a function of how quickly the dye can rotate during the lifetime of the excited state. Fluorescence lifetimes generally last a few nanoseconds. Small fluorophores in aqueous solutions, such as fluorescein, have rotational diffusion times in the hundreds of picoseconds. Extensive movement during the excitation state produces essentially isotropic fluorescence (i.e., photons of any geometry), indicated by a loss of measurable polarization. Large fluorescent proteins, on the other hand, rotate much more slowly and have rotational diffusion times that are roughly 10-fold greater than their fluorescence lifetimes [31,32,33]. Consequently, they do not move much during the fluorescence cycle, and the measured polarization of the emitted photons is very close to the photoselection plane. Fluorescent protein fluorescence, is thus, highly anisotropic.

The constraint placed by molecular size on fluorescent protein anisotropy is so strong that the only practical method to depolarize fluorescence is to transfer energy to a second molecule outside the photoselection plane through FRET (Figure 1c). Further, the amount of depolarization observed is strongly proportional to the amount, i.e., efficiency, of FRET. For example, shortening the distance separating two tandem fluorescent proteins increases depolarization through enhanced FRET [15,34]. The theory underlying these experimental observations is quite sound, as it was this relationship between depolarization and fluorophore distance/orientation that led to the discovery of FRET, and the derivation of the Förster equation [21,22].

### 2.2. FRET Efficiency and Depolarization

The “efficiency” of FRET between two fluorophores is the fraction of donor molecules that transfer energy to acceptors. There are several available methods for calculating two-color FRET efficiency, including fluorescence lifetime measurements of the donor [35,36], photobleaching methods that destroy acceptor fluorescence [15,37], and spectral imaging [38]. All of these approaches rely on the separate collection of donor and acceptor fluorescence, which can be challenging depending on the FRET pairing and the available optics.

Homotransfer FRET, however, can be quantified by measuring fluorescence polarization, since FRET efficiency is proportional to the depolarization of fluorescence emission. Polarizations are conventionally described by their anisotropy (r), which is defined by the difference between the two measured orientations, parallel (***P***) and perpendicular (***S***) intensity, over the total fluorescence:

(1) $$\;r=\frac{\left(P-gS\right)}{\left(P+2gS\right)}\;$$

where (g) corrects for polarization bias in the instrumentation. Förster [21] related changes in the degree of polarization with quantum yield according to:

(2)  ppmax=6n1n(5+n1n) 

where n is the total quantum yield of fluorescence,$\;n_{1}$ is the quantum yield of fluorescence of the donor molecules, p is the degree of polarization of the sample, and $p_{max}$ is the maximum degree of polarization in the absence of the energy transfer. The degree of polarization is defined by the ***P*** and ***S*** intensities:

(3) $$\;p=\frac{P-S}{P+S}\;$$

Fluorescence depolarization is related to the rate of energy transfer, $k_{ET}$, by way of quantum yield. The donor fluorophore’s quantum yield is dependent on the total quantum yield of both the donor and acceptor and the rate of energy transfer:

(4) $$\;n_{1}=n\frac{1+\tau k_{ET}}{1+2\tau k_{ET}}\;$$

The FRET efficiency, E, is defined as:

(5) $$\;E=\frac{\tau k_{ET}}{1+\tau k_{ET}}\;$$

where τ is the average lifetime of the excited state in the absence of an acceptor. Through substitution, Equations (2), (4), and (5) can be combined to give:

(6) $$\;E=\frac{6\left(p_{max}-p\right)}{5p}\;$$

Polarization can then be written in terms of anisotropy to arrive at the final equation:

(7) $$\;E=\frac{12\left(r_{max}-r\right)}{5r\left(2+r_{max}\right)}\;$$

The relationship between depolarization and the FRET efficiency is similar for heterotransfer. The FRET efficiency expressed in terms of anisotropy is thus:

(8) $$\;E=\frac{6\left(r_{max}-r\right)}{5\left(r+\frac{1}{2}\right)}\;$$

**Figure 2:**
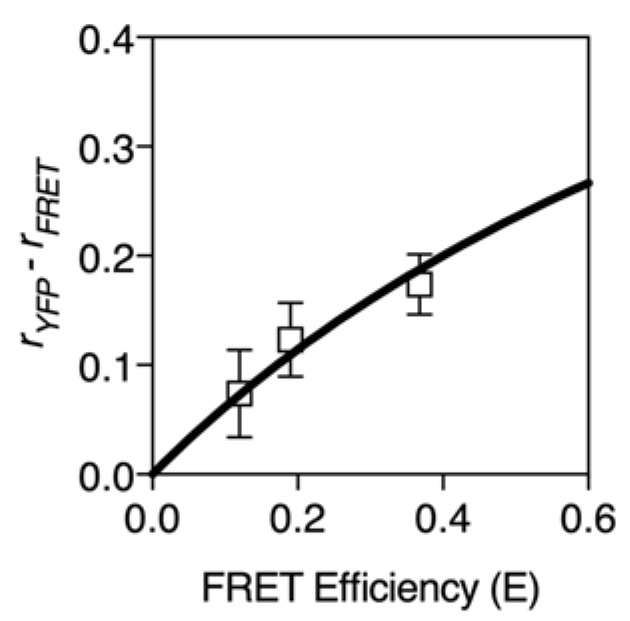
FRET efficiency vs. polarization. FRET efficiency increases with decreasing anisotropy. FRET efficiency for heterotransfer is plotted for various anisotropy values (*r* = 0 to *r* = 0.3) according to Equation (8). A maximum anisotropy of 0.3 is assumed. FRET values (mean ± S.D., *n* = 7 cells) from previously published standards [15,39] were quantified using fluorescence polarization microscopy.

Experimental FRET efficiency measurements for heterotransfer match this derivation reasonably well (Figure 2).

### 2.3. Calculating FRET Efficiency from Time-Resolved Anisotropy Measurements

Time-resolved anisotropy measurements can also be used to calculate homotransfer FRET efficiency. Based on the rate of fluorescence anisotropy decay, the FRET efficiency can be estimated, and it is used to calculate interfluorophore distances [40,41]. Furthermore, the average number of proteins in a cluster can be calculated from three additional parameters: the anisotropy of monomeric fluorophores, the anisotropy of fluorophores excited by energy transfer, and the steady-state anisotropy [42,43]. Though time-resolved anisotropy measurements permit the detailed study of molecular complexes, obtaining sufficient photons for computational analysis can be challenging [44].

## 3. Fluorescence Polarization Microscopy

### 3.1. Steady-State Polarization Measurements

Widefield systems can be configured for steady-state fluorescence polarization microscopy by the addition of plate polarizers into the optical pathway [27,45]. An excitation polarizer can be placed between the light source and the fluorescence filter cube. Depending on the microscope vendor, this can be within the microscope stand (e.g., Zeiss) or adjacent to the illumination source (e.g., Nikon). The orientation of the polarizer is generally fixed either horizontally or vertically to facilitate the capture of the required emitted light polarizations.

After the emitted light passes through the fluorescence filter cube, images containing parallel (***P***) and perpendicular (***S***) components may be simultaneously collected with a single camera using an image-splitting device. Polarization-compatible devices are available from Optical Insights (Dual-view), Hamamatsu (W-view Gemini), and Cairn (Opto-split), and we have implemented each of these solutions successfully. Alternatively, ***P*** and ***S*** images can be collected sequentially using a filter wheel to exchange suitably oriented polarizers.

### 3.2. Time-Resolved Measurements

Dynamic, time-resolved anisotropy can also provide information on FRET [32,46,47]. The decay in anisotropy over nanosecond timescales can be measured using pulsed excitation and ultrafast recording strategies, such as time-correlated single photon counting [48]. The resulting data is fit to a model that contains two main decay components: the slow decay component represents the rotational correlation time of the fluorophore, while the fast decay component represents the energy transfer due to FRET. Although the ability to separately quantify molecular rotation and FRET can be quite powerful, lifetime microscopy is a photon ‘hungry’ technique [49]. Collection of large numbers of photons necessitates tradeoffs, such as long integration times or low spatial resolution, which can limit live cell experimentation.

### 3.3. Optical-Sectioning

Optical-sectioning technologies, such as laser scanning confocal microscopy, can be challenging to configure for fluorescence polarization microscopy for several reasons. Fiber optics can degrade laser polarization if they are used to deliver light to the scanning unit. The collection of ***P*** and ***S*** images is also problematic, as many commercial sources do not presently offer configurations that incorporate plate polarizers into the emission pathway.

We have had more success configuring two-photon microscopes for polarization imaging [15,27,34]. The photon pulses needed for two-photon excitation necessitate free-space coupling of the laser to the microscope, which also preserves laser polarization. Further, fluorescence emission can be collected in a so-called non-descanned configuration that bypasses the scanning mirrors. This configuration is easily accessed and allows straightforward placement of the emission polarizers in front of the detectors [27].

Two-photon excitation is also more photo-selective, raising the measured anisotropy of fluorescent proteins from 0.3 to 0.4 [15]. On the other hand, the photoselection advantage can be theoretically offset in thick tissues by depolarization from light scattering [50]. Even so, several groups have successfully performed two-photon homotransfer imaging for specimens as varied as acute hippocampal brain slices [51], mouse skeletal muscle [34], and Drosophila larvae [51]. Light scattering in thick tissues has not yet been an insurmountable limitation.

While polarization microscopy is indeed compatible with conventional optical sectioning microscopy methods, such as confocal and two-photon, it is far from an optimal marriage of technologies. Information from ***P*** and ***S*** images are typically ratioed, which is particularly sensitive to low signal-to-noise [52]. Indeed, this necessitates long collection times, sometimes exceeding several minutes even in widefield, to generate images of sufficient quality for analysis. Consequently, sample photodamage can become limiting for polarization imaging, particularly during optical sectioning applications.

Newly developed optical sectioning approaches can improve image signal-to-noise and provide gentler sample illumination conditions. Inverted selective plane illumination microscopy (iSPIM) [53], which illuminates the sample with a light sheet positioned perpendicularly to the collection lens, has been successfully configured to accommodate polarization microscopy [45,54]. Using this method, we were able to perform homotransfer FRET biosensor imaging over a seven-hour period in developing *C. elegans* embryos [45]. Image collection speeds during this experiment approached video rate, which is at least 10 times faster than the previous optical sectioning methods that we have tested.

## 4. Biosensor Design

### 4.1. Double-Fluorophore Biosensors

The most straightforward method for constructing a homotransfer reporter is to convert a validated heterotransfer biosensor by swapping out the fluorescent proteins for a homotransfer pair. Calcium biosensors were among the first converted to homotransfer [34,55], but several others have since been adapted (Table 1), including the fluorescence anisotropy reporters, or FLAREs [34].

Several biosensors of varied design have been successfully converted to homotransfer reporters, demonstrating the robustness of the approach. Classic “molecular switch” type sensors, such as the Protein Kinase A activity reporters (AKARs) that contain both external sensing domains and internal effector-binding domains, have been converted to homotransfer reporters in four different colors [34]. Sensors that report changes in a full sequence protein, like myosin light chain kinase (MLCK) [34] or glucokinase [56], have also been successfully converted to FLAREs.

Homotransfer reporters can be optimized using the same strategies developed for heterotransfer reporters. One approach that is particularly noteworthy is the use of circularly permuted fluorescent proteins. Such variants are generated by fusing the natural N and C termini of the fluorescent protein, and creating alternate starting and ending positions in the fluorescent protein sequence [57]. Incorporation of circularly permuted fluorescent proteins effectively introduces an alternate chromophore position for the biosensor, which may improve the dynamic range of the sensor compared to ones containing the non-permuted variant.

Circularly permuted versions of the mCerulean3 CFP [59] and the mVenus YFP [60] have been used to successfully optimize several FLAREs, including AKAR4 [34]. Interestingly, the combination of mVenus and a circularly permuted mVenus has thus far been the most effective pairing for homotransfer reporters, as measured by the dynamic range [34]. The superior performance of mVenus variants in homotransfer reporters is likely to be related to the fluorescence properties of mVenus, which is both an excellent FRET donor because of its high quantum yield, and a superb FRET acceptor because of its high extinction coefficient [61].

### 4.2. Single-Fluorophore Biosensors

Biosensors can also be created using a single fluorescent protein label (Table 2) since biomolecules frequently form dimeric or multimeric complexes that bring them close enough to observe homotransfer. For membrane-associated molecules, like phospholipids, FRET can be used to detect clustering into subdomains [62]. Similarly, fluorescent protein markers of lipid subdomains [32] or phospholipid aggregation [63] also exhibit homotransfer in living cells. Fluorescence polarization has also been used to quantify the oligomerization state of membrane proteins, such as the epidermal growth factor receptors [64,65].

Conformational changes within protein complexes have also been resolved using homotransfer. Structural rearrangement of singly-labeled Ca^2+^/calmodulin-dependent protein kinase II has been observed during its activation using homotransfer [18]. Homotransfer can also be used to quantify green fluorescent protein (GFP)-labeled actin polymerization into fibers [51].

Single-color sensors can also be engineered using rational design. The Apollo nicotinamide adenine dinucleotide phosphate (NADP+) probe uses a catalytically inactive glucose-6-phosphate dehydrogenase mutant as the sensing domain [50]. Dimerization of singly-labeled subunits occurs as NADP+ rises and is fully reversible. A single fluorescent protein sensor for non-muscle myosin II was similarly created by targeting reversible dimerization. The regulatory light chain of myosin II naturally dimerizes until activated by phosphorylation. Fusion of this myosin subunit with a fluorescent protein permits detection of the phosphorylation event by monitoring homotransfer [45].

## 5. Comparison to Heterotransfer Reporters

### 5.1. Multisensor Applications

One of the principal advantages of employing homotransfer reporters is the ability to accommodate secondary fluorescence and optogenetics tools. It remains challenging to measure multiple two-color FRET sensors in the same cell at the same time. FRET reporters generally use either the cyan/yellow or green/red FP combinations. Since these pairs take up a broad swath of the useful visible light spectrum, pairing them with a second FRET reporter may require stretching into the near-infrared [66]. Further, the color combinations available for heterotransfer sensors are not very flexible. For example, the most prevalent heterotransfer pairs generally include a YFP or GFP, making them difficult to pair with secondary green indicators.

Homotransfer sensors, however, can be more easily switched to optically compatible areas of the spectrum. For example, the Apollo NADP+ sensor was converted from YFP to CFP for use with a green peroxide indicator [50]. Additionally, an mCherry-tagged phosphoinositide reporter was combined with a CFP/YFP heterotransfer FRET calcium sensor [63]. Our homotransfer myosin II reporter also shows similar flexibility. We paired a CFP version with red phosphoantibodies for colocalization experiments and then switched the sensor to mCherry for experiments with CFP/YFP heterotransfer biosensors [45]. Homotransfer sensors of various colors can also be combined for multiplexed experimentation. Oscillatory calcium and cyclic adenosine monophosphate circuits have been measured in individual pancreatic beta cells using compatible homotransfer FLAREs [34]. Proof of principle studies have shown that as many as three FLARE biosensors (CFP calcium sensor, YFP Mitogen-Activated Protein Kinase sensor, and mCherry Protein Kinase A sensor) can be used together in a single living cell. 

Intravital, multiplexed imaging of FLAREs has also been performed using two-photon microscopy [34]. The biosensor color combinations for two-photon imaging requires special consideration because the two-photon absorption spectra for fluorescent proteins is broader than for single-photon absorption [67]. Using a broadband fluorescence filter for collection, along with selective excitation conditions, we were able to exclusively excite a YFP FLARE calcium sensor or an mCherry FLARE AKAR sensor expressed together in a skeletal muscle preparation [34]. We observed independent activation of the sensors to either local electrical stimulation or systemically-administered isoproterenol [34].

### 5.2. Compatibility with Other Optical Tools

The spectral flexibility of homotransfer reporters is also advantageous for combination with optical reporters. Light-gated ion channels, such as the ChRs, tend to have absorbances that overlap with CFP, GFP, and YFP. For example, a commonly used ChR has a peak excitation at approximately 460 nm [68], making it optically incompatible with conventional CFP/YFP heterotransfer reporters. In contrast, red homotransfer sensors are compatible with ChR. We have even used a YFP FLARE to detect calcium responses during ChR activation [34].

The flexibility of the homotransfer sensors also permits them to be used with other optogenetic tools such the photo-inducible protein assembly using the light-oxygen-voltage-sensing (LOV) domain [69]. Fusion proteins containing LOV domains have been used to control DNA binding [70], enzyme activity [71], dimerization [72], and localization [73]. Despite improvements in photocycle lifetimes [74], LOV domains have limited capacity for color tuning. The flavin chromophore present in all LOV domains is small and rigid providing little room for adjustment. Similar to ChR, LOV domains absorb blue light with a peak absorbance at 450 nm [75]. Studies using LOV domains have previously incorporated red fluorescent protein reporters [66]. Thus, mCherry homotransfer reporters would also be compatible.

### 5.3. Quantitative Analysis

Despite less overlap between the donor and emission spectra, homotransfer reporters still compare favorably to heterotransfer biosensors in generating contrast. The signal-to-noise ratios of the sensors, as measured by the maximal change in response divided by the standard deviation of the basal state [59] ranges from a 5-fold to over a 30-fold change for FLAREs. Direct comparison to heterotransfer reporters shows that homotransfer can be 4–5 fold more efficient by this metric [56]. The improved contrast of homotransfer reporters primarily results from the increased precision of fluorescence polarization measurements [56].

The nature of the data collected for polarized FRET is also advantageous for quantitative analysis because channel crosstalk is eliminated. For heterotransfer FRET, acceptor excitation [15] and donor bleed-through into the acceptor channel [16,17] can both complicate FRET quantification. Single color studies do not require bleed-through correction, and they can also be normalized to the total fluorescence through the anisotropy calculation. Simplifying FRET quantification facilitates comparison between samples, even at the image pixel level.

We first used automated image analysis of homotransfer data to study protein-protein interactions in the endoplasmic reticulum (ER). The sarco(endo)plasmic calcium ATPase (SERCA) pumps calcium into the ER/sarcoplasmic reticulum lumen, and it is regulated by several small transmembrane peptides [76], including sarcolipin (SLN) and small ankyrin 1 (sAnk1), which form multimeric aggregates in the ER membrane. Association between sarcolipin and sAnk1 can be detected using a bimolecular complementation approach, in which a fluorescent protein sequence is split into two parts and used to label the proteins separately. Tagged SLN and sAnk1 give rise to a full fluorescent mVenus molecule when they associate, and multimerization results in homotransfer between the SLN–sANK1:mVenus complexes [77].

To examine the effect of SERCA interaction on SLN–sANK1:mVenus homotransfer across many cells, we developed a computationally automated analysis approach [77]. Previously, we calculated anisotropies from individual cells using hand-selected regions of interest. In contrast, our automated analysis extracted pixel anisotropies from approximately one hundred cells and pooled the data together. The resulting data set contained information from ~10,000 pixels and revealed that SERCA could affect the structure of the SLN–sANK1:mVenus multimers [77].

We used the automated pixel-by-pixel analysis in combination with a two-fluorescent protein biosensor for glucokinase activity [56], which undergoes a complex series of conformational transitions when it becomes activated. Several different pathways can lead to glucokinase activation. Even so, the relationship between the different post-translationally activated conformational states has been challenging to understand, particularly in living cells. We used a homotransfer reporter for glucokinase and pixel level quantitative analysis to show that the different activation mechanisms induce a singular activated state. Information from hundreds of images was extracted and sorted using the R statistical computing package and EBImage [78]. Anisotropy values from approximately 100,000 pixels per group were compiled for comparison, permitting a quantitative assessment of the FRET distributions for different glucokinase conformations in living cells.

We performed a similar analysis to track myosin phosphorylation in developing *C. elegans* [45]. Here, we knew the number of sensor conformational states but needed to calculate the anisotropies of the two states from iSPIM data sets, comprised of 60 optical sections taken every 5 min over seven hours. Analysis of anisotropies at the pixel level not only provided the volume of information needed to fit the distribution of values to a two-state model but also greatly facilitated the speed of analysis. Handling data at the pixel level allows us to bypass the computational load required to display the data set graphically.

Large-scale pixel level analyses are much more difficult to apply to heterotransfer FRET data, at least in our experience. Although numerous corrective methodologies [16,79,80] have been devised to handle the bleed-through problem, we have not found a satisfactory method for treating the error in the corrective factor measurements. Further, the intensity values used for heterotransfer calculations are inherently noisier, frequently necessitating data reduction methods, such as baseline normalization and reliance on means. Our application of homotransfer to the glucokinase problem was our solution to the technical difficulties that arose while using heterotransfer glucokinase reporters.

**Figure 3:**
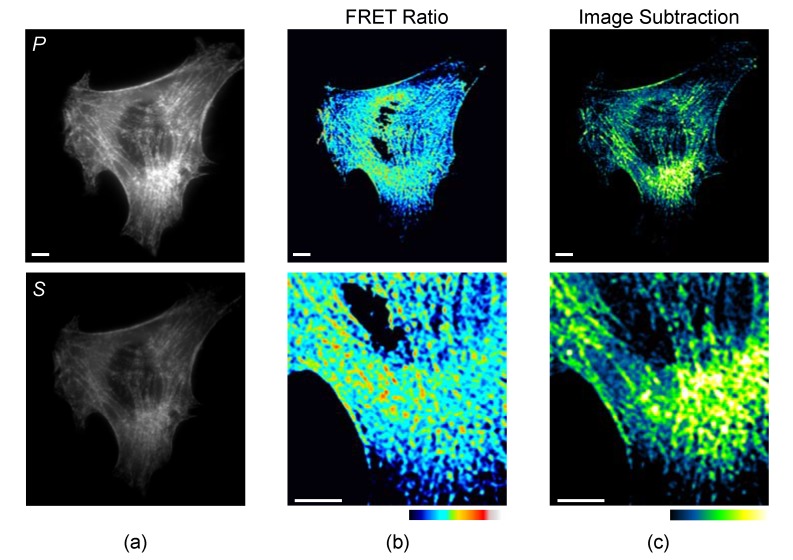
Image analysis of an NIH3T3 fibroblast expressing the mCerulean3 myosin II biosensor [45]. (**a**) ***P*** and ***S*** images were collected using widefield polarization microscopy; (**b**) the images can be ratioed (***P***/***S***) using a conventional processing strategy. Bottom shows an enlarged area of the lower perinuclear region; (**c**) alternatively, a scaled ***S*** image can be subtracted from the ***P*** image to preserve differences in sensor localization that are obscured by the intensity-independent image ratio method. Scale bars are 5 μm.

Fluorescence polarization microscopy also provides an alternative for FRET image representation (Figure 3). Typically, donor and acceptor images (or ***P*** and ***S***, Figure 3a) are ratioed to generate a map of intracellular FRET dynamics. While ratio images are a generally useful approach, the loss of intensity information is disadvantageous if the sensor is compartmentalized. For example, Figure 3b shows a FRET ratio image for the myosin II FRET sensor in a fibroblast. The ratio image neutralizes intensity differences, which obscures compartmentalization on fibers, particularly in and around the nucleus (Figure 3b, bottom panel).

Intensity information can be preserved in polarization FRET images using image subtraction, rather than image ratioing. Since the ***S*** channel is isotropic by definition, it can be subtracted from the ***P*** image to highlight regions with the most polarization. Further, the ***S*** channel can be multiplied by a normalization factor to mask areas of less than the desired anisotropy threshold [45]. Normalized image subtraction using this method can be useful for sensors like the myosin II sensor (Figure 3c) that become compartmentalized upon activation.

## 6. Conclusions

Homotransfer reporters offer significant advantages over heterotransfer biosensors, including increased measurement precision, flexibility for multiplexing, and improved methods for data analysis. Furthermore, a large number of validated heterotransfer biosensors have been converted to homotransfer reporters, and they are readily available for biological experimentation. Recent advances in data analysis and optical sectioning have also expanded the power of the FRET-based biosensor approach and can now be used to explore biological questions in complex physiologic systems.

## Figures and Tables

**Table 1:** Double-fluorescent protein homotransfer reporters.

Sensor	Color	Reference
Protein Kinase A (AKAR)	cyan fluorescent protein (CFP), yellow fluorescent protein (YFP), green fluorescent protein (GFP), mCherry	[34]
Cyclic adenosine monophosphate (ICUE3)	YFP	[34]
Calcium (Cameleon)	CFP, YFP, mCherry	[34,55]
Calcium (Twitch-4)	YFP	[58]
ER Calcium (D1)	CFP	[34]
Myosin Light Chain Kinase	YFP	[34]
Protein Kinase C (CKAR)	CFP, YFP, mCherry	[34]
Mitogen-Activated Protein Kinase (EKAR)	CFP, YFP, mCherry	[34]
Glucokinase	YFP	[56]

**Table 2:** Single-fluorescent protein homotransfer reporters.

Sensor	Color	Reference
Actin	GFP	[51]
Non-muscle myosin II	CFP, GFP, mCherry	[45]
Ca^2+^/calmodulin-dependent protein kinase II	YFP	[18,47]
Akt pleckstrin homology domain	mCherry	[63]
glycophosphatidylinositol	GFP	[32]
Epidermal growth factor receptor	GFP	[65]
Nicotinamide adenine dinucleotide phosphate (Apollo)	CFP, YFP	[50]
